# Violations in the marketing of milks and complementary foods that compete with breastfeeding in Rio de Janeiro City, Brazil

**DOI:** 10.1590/1984-0462/2023/41/2021228

**Published:** 2022-07-06

**Authors:** Lucilene Antônio Afonso Bertoldo, Maria Inês Couto de Oliveira, Cristiano Siqueira Boccolini

**Affiliations:** a Fundação Oswaldo Cruz, Rio de Janeiro, RJ, Brazil.; b Universidade Federal Fluminense, Niterói, RJ, Brazil.

**Keywords:** Breast feeding, Breast-milk substitutes, Direct-to-consumer advertising, Legislation, food, Products commerce, Aleitamento materno, Substitutos do leite humano, Publicidade direta ao consumidor, Legislação sobre alimentos, Comercialização de produtos

## Abstract

**Objective:**

To analyze if milk and complementary foods are being sold under the Brazilian Code of Marketing of Infant and Toddler's Food, Teats, Pacifiers and Baby Bottles (NBCAL), Law 11265/2006 of breastfeeding protection.

**Methods:**

Epidemiological survey that analyzed the marketing practices of pharmacies, supermarkets, and department stores in the Southern region of the city of Rio de Janeiro, Brazil, by direct observation.

**Results:**

Among the 349 stores in Rio de Janeiro's South Region, 339 traded milk and complementary foods and, among them, 60.8% were not complying with NBCAL. Infractions to NBCAL were more common for the selling of milk (58.6%) than complementary foods (22.8%). The most recurrent promotion strategy infringing NBCAL was discount pricing without the Ministry of Health disclaimer.

**Conclusions:**

Most retail stores infringe NBCAL in the commercialization of milk and complementary foods in the city of Rio de Janeiro, Brazil, a violation of the right to information that may impact mothers’ choice regarding their child's feeding.

## RESUMO

**Objetivo:**

Analisar se leites e alimentos de transição estão sendo comercializados de acordo com a Norma Brasileira de Comercialização de Alimentos para Lactentes e Crianças de Primeira Infância, Mamadeiras, Bicos e Chupetas (NBCAL), Lei n. 11.265/2006, de proteção ao aleitamento materno.

**Métodos:**

Inquérito epidemiológico que analisou as práticas de promoção comercial de leites e alimentos de transição em um censo de farmácias, supermercados e lojas de departamento da Zona Sul da cidade do Rio de Janeiro, por meio de observação direta.

**Resultados:**

Dos 349 estabelecimentos da Zona Sul, 339 comercializavam leites e alimentos de transição e, destes, 60,8% faziam promoção comercial em desacordo com a NBCAL. Mais da metade dos estabelecimentos (58,6%) tinha leites e 22,8%, alimentos de transição vendidos em inconformidade com a NBCAL. A estratégia de promoção comercial mais praticada foi o desconto no preço sem a frase informativa preconizada pelo Ministério da Saúde.

**Conclusões:**

A maior parte dos estabelecimentos comerciais infringe a NBCAL na comercialização de leites e alimentos de transição, configurando uma violação ao direito à informação que pode impactar na escolha das mães quanto à alimentação de seus filhos.

## INTRODUCTION

The United Nations (UN) declared in 2016 that breastfeeding is a human right for both children and mothers, and recommended that abusive commercial practices of breastmilk substitutes be curbed.^1^ The Nutrition Guide for Children Under the Age of Two recommends exclusive breastfeeding until 6 months of life and the continuation of breastfeeding until 2 years or more, complemented with foods prepared by the family, respecting cultural and food identity of different regions and excluding industrialized food.^2^ However, the feeding of infants and young children has been the target of abusive marketing with illegal commercial promotions of milk and processed complementary food.^3–5^

Between 2008 and 2013, annual global sales of so-called breast milk substitutes increased by 40.8%, from 5.5 to 7.8 kg per child, with a forecast that in 2018 they would reach 10.8 kg per child per year.^6^ In monetary figures, sales of breastmilk substitutes worldwide reached 44.8 billion dollars in 2014, with an estimate of reaching 70.6 billion dollars in 2019.^7,8^

The Brazilian Code of Marketing of Infant and Toddler's Food, Teats, Pacifiers and Baby Bottles (NBCAL) is a legal mechanism that regulates commercial promotion and labeling of foods and products intended for newborns and children under 3 years of age in Brazil.^9^ The NBCAL was created in 1988 through Resolution No. 5 of December 20, based on the International Code of Marketing of Breast Milk Substitutes, having undergone updates in 1992, 2001 and 2002, being strengthened as Law 11.265/2006^9^ and regulated in 2015 and 2018.^10^

The NBCAL defines commercial promotion as a “set of informative and persuasive activities coming from companies responsible for production or manipulation, distribution and commercialization aiming to induce the purchase or sale of a certain product”.^9^ Any type of commercial promotion of teats, pacifiers, baby bottles, infant formulas and follow-up infant formulas is prohibited in this setting. Commercial promotion of milk is allowed, as long as it displays the phrase “The Ministry of Health informs: breastfeeding prevents infections and allergies and is recommended until two years of age or more”; the same applies to processed complementary food, whose promotion must display the sentence “The Ministry of Health advises: after six months of age, continue to breastfeed your child and offer complementary foods”.^9^

Studies conducted in different Brazilian settings have indicated that the NBCAL is not being adequately complied with.^11–13^ The objective of this study was to assess whether milk and processed complementary food are being marketed in accordance with this law, focusing on contributing to its compliance.

## METHOD

This is an epidemiological survey, part of the research “Evaluation of the compliance with the Brazilian Code of Marketing of Infant and Toddlers Food in Commercial Establishments”, which included a census of commercial facilities (pharmacies, supermarkets and department stores) in the South Region of the city of Rio de Janeiro, which was selected for holding a population from various social segments (low, medium and high income) distributed across 18 neighborhoods^14^ of the upper class, such as Leblon and Ipanema, and low-income, such as the communities of Rocinha and Vidigal, which are characterized by irregular soil and poor urban infrastructure.^15^

In this study, an initial list of commercial facilities was created by consulting the *Tele-Listas*, and with address information obtained from the websites of the main commercial networks. For data collection, all supermarkets, pharmacies and department stores located in the selected region were visited; those that were not included in the previous list and were found by the interviewers when commuting through neighborhoods were also added to the study. Manipulation or homeopathic pharmacies, establishments that did not sell products under the scope of NBCAL or did not have a registry number (CNPJ) were excluded.

All data were collected between March and April 2017 by seven health professionals previously trained in the NBCAL monitoring course, supervised by two research coordinators. A questionnaire adapted from the International Baby Food Action Network (IBFAN-Brasil) was created for the study. The questionnaire was inserted into the mobile data platform Magpi (2018), commonly used in studies in the health field.^16^ Cell phones and tablets with the Magpi application installed were used to apply the questionnaires.

First, the commercial facility (pharmacy, supermarket, department store) was identified. Upon entering the establishment, the interviewer identified the groups of products sold at the location that were covered by the NBCAL and kept a record of whether there were commercial promotions that infringed the rules for each product. Then, the managers of the commercial establishments were interviewed upon presentation of the approval by the Ethics Committee and after their verbal consent.

For the present study, the commercialization of two major groups of products covered by the NBCAL was evaluated: milks and complementary foods, as well as their commercial promotion practices, as regulated by the NBCAL.

Because dairy compounds are marketed with the indication of use “from 1 year of age” (observed in all products in this category), they were considered as “Breastmilk substitute (BMS)”, being “ any food being marketed or otherwise presented as a partial or total replacement for breastmilk”.^9^ Dairy compounds were, therefore, allocated in the milk group, since Decree n. 9,013 of 2017 defines them as “powdered dairy product obtained from milk or milk derivatives or both”.^17,18^ “Modified, fluid or powdered milk are dairy products resulting from the modification of milk composition by subtracting or adding its constituents.”^17^ Processed complementary foods are defined as industrialized products for direct use or in homemade preparations as a complement to breastmilk or to modified milk introduced in the diet of infants and toddlers.^9^

In order to assess compliance with the NBCAL, the following commercial promotions, defined by Law n. 11.265/2006, were checked:^9^ “special display: any way of exposing a product to make it stand out in the commercial establishment, such as a showcase, special shelf positioning, island stacking, crates, ornamentation”; practice of discounts (individual, progressive, two for the price of one or use of discount coupons); and offer of gifts in the purchase of products.

The outcome of the study was illegal marketing of milk and/or complementary foods, characterized by the presence of commercial promotion without the corresponding disclaimer from the Ministry of Health or with illegible wording, without a frame, characters not in capital letters and bold, distant or in a vertical position in relation to the product, which constitutes infringement, as recommended by NBCAL.^9^

The following characteristics of commercial establishments were evaluated: segment (pharmacy, supermarket or department store), belonging to a network, and location (neighborhood or community). In addition, the promotion strategies of each product were analyzed, with focus on the following categories:

- Special display.
- Price discount.
- Giveaway.

As NBCAL infraction can be present in more than one promotion strategy in the same establishment, they were categorized into:

- 0: no promotion.
- 1: special display.
- 2: price discount.
- 3: giveaway.
- 4: special display and price discount.
- 5: price discount and giveaway.
- 6: special display and giveaway.
- 7: all strategies.

The data were analyzed in the Statistical Package for the Social Sciences (SPSS), VERSION 17. Descriptive analyzes were performed, characterizing the types of establishments, whether they belonged to a network and location. The frequencies of commercial establishments that sold milk, dairy compounds and complementary foods were also presented by type. The frequencies of pharmacies, supermarkets and department stores with commercial promotions of milk, dairy compounds and complementary foods in disagreement with the NBCAL were checked and categorized by type of commercial promotion strategy. Finally, the proportion of commercial establishments violating the NBCAL was assessed by product group, and the percentage of commercial establishments complying with NBCAL for the commercialization of milk and complementary foods was analyzed by segment.

This study followed the resolutions 466/2012 and 510/2016 by the National Health Council and was approved by the Research Ethics Committee of Hospital Universitário Antônio Pedro, Universidade Federal Fluminense (UFF), under opinion 1,878,013/2016. The research was funded by the National Council for Scientific and Technological Development (CNPq) and the Foundation for Research Support of the State of Rio de Janeiro (FAPERJ). We were granted exemption from the consent and signing a free and informed consent form by the commercial establishments and managers responsible for them, as this procedure could expose them to sanctions by the companies. Previous request for authorization at the commercial establishments could lead the person responsible to change the environment before data collection, by removing of illegal products and promotions to comply with the law.

## RESULTS

In this study, 349 commercial establishments were evaluated, of which 10 were excluded from the analysis (six pharmacies and four stores) for not selling milk or complementary foods. Thus, a total of 339 commercial establishments were part of the analysis, of which 68.1% were pharmacies and 78.2% belonged to a network, with the highest concentration of commercial establishments located in neighborhoods (Table 1).

**Table 1:** Characterization of commercial establishments per segment, network and location. South Region of the city of Rio de Janeiro, 2017.

Characteristics		n	%
Type of establishment			
	Pharmacy	231	68.1
	Supermarket	88	26.0
	Store	20	5.9
Belongs to a network			
	Yes	265	78.2
	No	74	21.8
Location			
	Neighborhood	314	92.6
	Community	25	7.4
	Total stablishments	339	100.0

**Figure 1:**
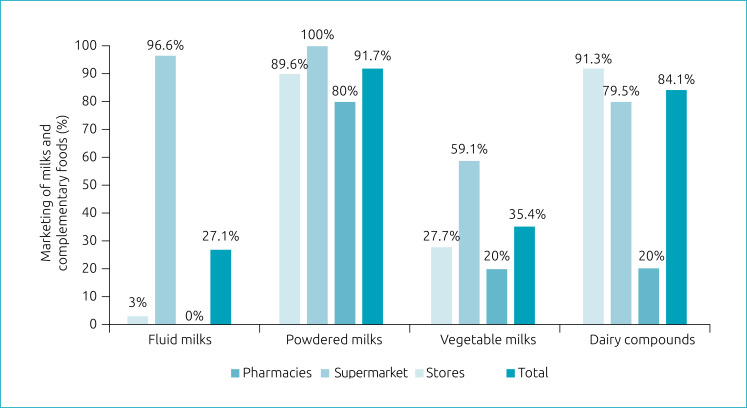
Marketing of milks and complementary foods by type of establishment. South Region of the city of Rio de Janeiro, 2017.

All supermarkets analyzed and 80% of department stores sold powdered milk; none of the stores sold fluid milks. Almost all pharmacies sold complementary foods. Powdered milk was the most commercialized product (Figure 1).

Considering all commercial establishments that had commercial promotions of milk and complementary foods in disagreement with the NBCAL (n=206), 84.9% of them used the strategy of price discount alone or combined with another type of promotion, followed by special display, in 52.9% of commercial establishments. Among pharmacies, the most commonly commercial strategy practiced in disagreement with NBCAL was special display combined with price discount, and among supermarkets and stores, price discount (Table 2).

**Table 2:** Commercial promotion strategies that do not comply with the Brazilian Code of Marketing of Infant and Toddler's Food, Teats, Pacifiers and Baby Bottles in the commercialization of milk and complementary foods by type of establishment. South Region of the city of Rio de Janeiro, 2017.

Commercial promotion strategy	Pharmacies		Supermarket		Stores		Total	
	n	%	n	%	n	%	n	%
Discount	41	28.7	34	60.7	7	100.0	82	39.8
Special display	19	13.3	4	7.1	0	0.0	23	11.2
Giveaway	6	4.2	0	0.0	0	0.0	6	2.9
Special display and discount	51	35.7	15	26.8	0	0.0	66	32.0
Discount and giveaway	9	6.3	0	0.0	0	0.0	9	4.4
Special display and giveaway	2	1.4	0	0.0	0	0.0	2	1.0
Special display, giveaway and discount	15	10.5	3	5.4	0	0.0	18	8.7
Total	143	100.0	56	100.0	7	100.0	206	100.0

The proportion of commercial establishments that committed some type of infraction in the marketing of milk was 58.6%, and in the marketing of complementary foods, 22.8% (Table 3).

**Table 3:** Proportion of establishments violating the Brazilian Code of Marketing of Infant and Toddler's Food Teats, Pacifiers and Baby Bottles for the sale of milk and complementary foods by type of establishment. South Region of the city of Rio de Janeiro, 2017.

Violation	Pharmacies		Supermarket		Stores		Total	
	n	%	n	%	n	%	n	%
Milks	134	59.0	54	61.4	7	38.9	195	58.6
Total	227	100.0	88	100.0	18	100.0	332	100.0
Complementary foods	62	28.4	9	10.3	0	0.0	71	22.8
Total	218	100.0	87	100.0	6	100.0	311	100.0

When analyzing compliance with the NBCAL by type of commercial establishment, only 7.6% of the 223 establishments offering promotions strategy complied with the NBCAL (Table 4).

**Table 4:** Compliance with the Brazilian Code of Marketing of Infant and Toddler's Food Teats, Pacifiers and Baby Bottles for the sale of milk and complementary foods by type of establishment. South Region of the city of Rio de Janeiro, 2017.

Compliance with NBCAL	Pharmacies		Supermarket		Stores		Total	
	n	%	n	%	n	%	n	%
Promotion not in compliance	143	96.6	56	82.4	7	100.0	206	92.4
Promotion in compliance	5	3.4	12	17.6	0	0.0	17	7.6
Total of establishments with promotions	148	100.0	68	100.0	7	100.0	223	100.0
Commercialization with no promotion	83	35.9	20	22.7	13	65.0	116	34.2
Total of establishments	231	100.0	88	100.0	20	100.0	339	100.0

NBCAL: Brazilian Code of Marketing of Infant and Toddler's Food Teats, Pacifiers and Baby Bottles

## DISCUSSION

Almost two thirds of the establishments that sold milk and complementary foods in the South Region of Rio de Janeiro violated the NBCAL, which jeopardizes the autonomy of mothers to choose the best way to feed their children. Breastfeeding and healthy complementary feeding are fundamental human rights to be protected;^19^ illegal marketing practices violate this right^7^ and can contribute to the reduction of breastfeeding and inadequate introduction of complementary feeding, compromising the development of infants.^20,21^

Studies have shown that mothers exposed to commercial promotions of milk and complementary foods in various media such as radio, television, printed materials and commercial establishments, and who remembered the promotional information are more likely to offer these foods to their children,^22–24^ which reinforces the importance of regulating commercial promotions of milk and complementary foods.

NBCAL exists for more than 30 years^3^ and it is an important tool to protect breastfeeding practice and a healthy complementary feeding, having undergone several improvements and gained the status of national law in 2006.^9^ The creation of NBCAL limits economic liberalism and should inhibit abusive marketing practices.^25^ However, the results of our study point to insufficient compliance with the law by commercial establishments.

The results indicate possible market segmentation and different dynamics in the commercial promotion of milks and complementary foods. The frequency of NBCAL infractions was high and similar between supermarkets and pharmacies and lower in department stores when selling milk. Almost a third of pharmacies, about a tenth of supermarkets and no department stores violated the NBCAL in the marketing of complementary foods. A study carried out with 25 supermarkets of Mossoró, Rio Grande do Norte, found, otherwise, that 64% of these establishments infringed the NBCAL when selling complementary foods and 16% when selling milk.^11^

The Ministry of Health recommends different informational sentences for milk and complementary foods. The sentence concerning dairy products informs about the superiority of breastfeeding in the prevention of infections and allergies, encouraging its practice. The informational line referring to complementary foods recommends offering new foods from six months of age on. The illegal commercial promotion occurred both due to the absence of the informative sentence and its inadequacy, when present, infringing Law n. 11,265/2006.

It is worth noting that only 7.6% of the commercial establishments offering commercial promotion of milk and complementary foods were complying with NBCAL, using the appropriate informational sentence from the Ministry of Health. Despite the low percentage of adequate commercial promotions found, it indicates that its practice in accordance with the law is possible. It is the obligation of establishments to know and comply with NBCAL, regardless of inspection by government agencies and the monitoring carried out periodically by organized civil society.^26^ However, the knowledge of establishments’ managers about the law is insufficient, as shown in a study that also used data of the survey “Evaluation of the compliance with the Brazilian Code of Marketing of Infant and Toddler's Food in Commercial Establishments”. Of the 309 managers of commercial establishments interviewed, 50.8% said they did not know the NBCAL, 24.3% mentioned having heard about it, and only 24.9% said they were familiar with this law.^14^

The periodic monitoring of the NBCAL carried out by organized civil society, such as the International Baby Food Action Network (IBFAN), has contributed to the enforcement of the law, but has been carried out in a non-representative sample of commercial establishments.^26^

Marketing must be ethical and responsible, not leaving aside what is recommended by current legislation,^25^ preventing mothers or guardians from being exposed to biased information that contributes to the acquisition of products by stimulating commercial promotion. However, in this study, commercial establishments that did not comply with the NBCAL, offering price discount without the appropriate informational line from the Ministry of Health highlighted was the most common strategy, both in isolation and in association with special display. The strategy of offering giveaways was the least frequent, observed in few pharmacies.

Infractions to NBCAL are subject to progressive penalties provided for in Law no. 6,437/77, which may lead to seizure of products, fines and establishment prohibition.^9^ However, where all segments of commercial establishments visited showed considerable percentages of illegal commercial promotions of milk and complementary foods, either these penalties have not had any effect or inspection is not being effective. The inspection of compliance with NBCAL is the responsibility of the Sanitary Surveillance,^3^ but there is a lack of periodic monitoring, which may prevent the increase in compliance with the law.^5^

The cross-sectional design of this study is one of its limitations, as it does not allow monitoring issues such as the seasonality of milk, which can generate greater sales stimulus when production increases and influence commercial promotion practices. Another limitation was the placement of dairy compounds in the same category as milk, since it is an ultra-processed product with its own characteristics, that is, added with industrialized substances extracted or refined from foods, usually oils, sugars and food additives.^2^ However, we proceeded this way because the Ministry of Agriculture and Livestock requires the presence of the same informative line in the labeling of both dairy compounds and milks.^17^

In addition, we did not verify whether there is a difference in compliance with the NBCAL for promotions of milk and complementary foods according to the location of the establishments: neighborhoods or communities. However, another study from the research “Evaluation of the compliance with the Brazilian Code of Marketing of Infant and Toddler's Food in Commercial Establishments”, through a logistic regression model, analyzed the factors associated with non-compliance with the NBCAL in the South Region of the city of Rio de Janeiro and reported no association between non-compliance with the law and the location of commercial establishments.^15^ Finally, it is worth mentioning that the present study evaluated compliance with the NBCAL in the commercialization of milk and complementary foods in the South Region of Rio de Janeiro, and the extrapolation of these results to other regions of the city or to other municipalities is limited.

Breastfeeding is one of the practices that most contribute to health in the world,^7^ and healthy complementary feeding contributes to the growth and development of the child and to the creation of eating habits that can persist into adulthood.^2^ Therefore, its protection against illegal commercial promotions that may influence the choices of parents and guardians on infant feeding becomes essential,^27,28,2^ through the publication of the Ministry of Health's statements about breastfeeding being recommended up to 2 years of age or more, since it plays a role in preventing infections and additional foods should be offered only after six months of age.

Conclusion is that there is non-compliance with the NBCAL in the commercialization of milk and complementary foods in commercial establishments in the South Region of Rio de Janeiro. The violation of the NBCAL in the commercialization of these products can impact the choice of mothers or guardians for the feeding of their children, constituting a violation of the right to information. Recently, the UN declared that breastfeeding is a human right for both children and mothers.^1^ Therefore, the widespread infringement of NBCAL violates human rights. On such occasion, it was recommended that states would make efforts to support and protect breastfeeding, as well as curb abusive commercial practices of breastmilk substitutes, ensuring that women have access to adequate information about the benefits of breastfeeding.^1^

The enforcement of law surveillance by competent bodies is recommended so as to comply with the NBCAL. Health Surveillance agents must be therefore trained in this regard, as well as investments must be made in their infrastructure. In addition, a more comprehensive knowledge and awareness of society about the importance of NBCAL is needed, which can be achieved by fulfilling one of the requirements of the law itself, which emphasizes the role of public bodies in educating and informing the society about NBCAL.

A review of the informational sentence to be published along with commercial promotions of complementary foods is suggested, as the line currently recommends offering new foods from six months of age on, without providing guidance on which foods these would be, allowing the industry to promote their products as appropriate. The informational line that accompanies complementary foods should be based on the recommendations of the Food Guide for Brazilian Children Under Two Years, which guides the introduction of in-natura or minimally processed foods in infant feeding from the beginning of complementary feeding, not recommending industrialized foods.^2^

Further studies that analyze the impact of promotions in commercial establishments on the choices of breastmilk substitutes are also suggested, as well as studies that evaluate the effectiveness of the NBCAL informational sentences as a mechanism of information for buyers. Finally, further studies are suggested in other regions of Rio de Janeiro and Brazil to assess differences in NBCAL compliance in other scenarios, taking into account the location of commercial establishments.
